# Targeted Lipidomics Insights into Lipid Discrepancies in Chicken Embryonic Brains Under Hypoxia Based on UHPLC-QTRAP-MS

**DOI:** 10.3390/ani16213515

**Published:** 2026-11-06

**Authors:** Qiao Xu, Xuewen Chai, Wangqin Liu, Jianning Huang, Danqin Tu, Haobin Wu, Qiguo Tang

**Affiliations:** 1 Jiangxi Provincial Key Laboratory of Poultry Genetic Improvement, Institute of Biological Technology, Nanchang Normal University, Nanchang 330032, China; 2 Development Center of Science and Technology, Ministry of Agriculture and Rural Affairs (MARA), Beijing 100176, China

**Keywords:** chicken, embryonic brain, lipidomics, hypoxic, lipid composition

## Abstract

Domestic chicken is an ideal model to study the relationship between lipid metabolism and environmental stress. However, the lipid composition of chicken embryonic brain under hypoxic conditions has not been systematically elucidated. We employed targeted lipidomics to comprehensively describe the alterations in the brain lipid profiles of embryonic day 18 TBCs and DLCs under hypoxia (13% O_2_, HTBC18, and HDLC18) and normoxia (21% O_2_, NTBC18, and NDLC18). Totally 1288 lipid species from 18 lipid classes were identified and screened for 47 (three up-regulated and 44 down-regulated) and 55 (10 up-regulated and 45 down-regulated) SDLs in HTBC18 compared to NTBC18 and HDLC18 compared to NDLC18, respectively. The five SDLs were shared between HTBC18 vs. NTBC18 and HDLC18 vs. NDLC18, including PI (16:0_20:3), PE (18:1_20:4), PC (20:0_22:5), PS (16:1_18:0) and PS (16:0_20:3), which were identified as candidate hypoxia-responsive lipids. Furthermore, the analysis of SDLs’ metabolic pathways revealed that glycerophospholipid metabolism was the most crucial. The present results provide insights into the dynamic composition of lipid in chicken embryo brains under hypoxic conditions.

## Simple Summary

Hypoxia is a critical environmental stressor impairing poultry embryonic development, and the lipid regulatory mechanism underlying hypoxic responses in the embryonic chicken brain remains poorly understood. Here, we performed targeted lipidomics to comprehensively analyze brain lipid profiles of 18-day-old embryos from Tibetan chickens and dwarf laying chickens under normoxic and hypoxic conditions. A total of 1288 lipid molecules belonging to 18 lipid subclasses were identified, and 47 and 55 significantly differentially abundant lipids (SDLs) were screened in the two chicken breeds under hypoxia, respectively. Five common SDLs were recognized as potential hypoxia-responsive lipids in chicken embryonic brains. Metabolic pathway analysis further revealed that glycerophospholipid metabolism dominates the embryonic brain’s hypoxic adaptation, accompanied by glycosylphosphatidylinositol anchor biosynthesis and autophagy processes. These findings characterize hypoxia-associated lipid profiles in the embryonic avian brain and provide a basis for further screening of potential lipid markers.

## 1. Introduction

Environmental stressors, including high altitude, high temperature, aridity and cold, serve as important driving forces for the adaptive evolution of organisms, which induces alterations in morphological structure, physiological function and molecular genetics, and further contributes to the formation of unique environmental adaptation characteristics [1,2,3]. Hypoxia represents one of the most important stressors in high-altitude environments, and the adaptive mechanisms adopted by high-altitude indigenous animals to cope with hypoxic conditions have long been a research hotspot in evolutionary biology. Early research on high-altitude hypoxic stress mainly focused on human populations [4,5,6]. In recent years, studies concerning hypoxic stress of other species sympatric with plateau indigenous human populations, such as Tibetan chickens, Tibetan pigs and yaks, have proliferated [7,8,9,10,11].

Compared with humans and other high-altitude mammals, chicken embryos exhibit independent in vitro incubation and developmental characteristics. The chicken embryo incubation process allows for precise regulation of oxygen concentration, which makes them an ideal model for exploring the regulatory mechanisms of high-altitude hypoxia adaptation in poultry. Current research on high-altitude hypoxia adaptation in chickens mainly focuses on genomic screening [12,13,14], transcriptomic and proteomic regulation [15,16,17], glycolytic metabolism [18] and mitochondrial function [19], while systematic investigations on lipid metabolism remain insufficient [20]. As essential structural components of neural cell membranes, lipids also participate in oxidative stress response, energy redistribution and apoptotic regulation, thereby playing vital roles in environmental adaptation [21]. Human high-altitude exposure studies have verified that lipid metabolism represents one of the core metabolic alterations under hypoxic conditions [22]. The brain is extremely sensitive to oxygen fluctuation, and hypoxia can trigger membrane component remodeling, lipid metabolism reprogramming and altered sphingolipid signaling in neural tissues. Mammalian brain studies have further confirmed that hypoxia significantly disturbs the homeostasis of glycerophospholipids, sphingolipids and lipid droplets in the hippocampus and cerebral cortex, indicating lipid remodeling as a conserved strategy for the central nervous system to cope with hypoxic stress [23,24]. With the rapid development of lipidomics, lipid metabolism has been widely recognized as a crucial adaptive mechanism for local high-altitude livestock against hypoxic injury. Nevertheless, the comprehensive brain lipid profiles of high-altitude and low-altitude chicken embryos under hypoxic and normoxic conditions remain unclarified, which limits our systematic understanding of the molecular mechanisms underlying chicken embryonic hypoxia adaptation at the lipid metabolomic level.

Lipidomics represents an important branch of metabolomics. Compared with other metabolites, lipids exhibit unique structural diversity and functional specificity, and participate extensively in biological processes including membrane formation, signal transduction, energy storage and stress response [25,26]. Driven by rapid advances in mass spectrometry, lipidomics has become one of the fastest-growing research fields in systems biology and attracted extensive attention from interdisciplinary researchers. The mainstream analytical techniques for lipidomics include shotgun lipidomics, nuclear magnetic resonance spectroscopy, gas chromatography–mass spectrometry and LC-MS. Among these approaches, LC-MS/MS-based lipidomics enables effective identification of low-abundance lipid species and supports high-throughput qualitative and quantitative profiling of hundreds of lipids [27,28]. This strategy has been widely adopted to characterize lipid compositions in chicken-derived samples such as muscle [29,30,31], eggs [32,33], liver [34,35] and brains [20]. However, few studies have evaluated lipids in embryonic chicken brains and their relationship with hypoxia using a targeted lipidomics approach.

This study employed targeted lipidomic techniques to investigate the changes in brain lipid profiles of Tibetan chickens and dwarf laying chickens at embryonic day 18 under hypoxic and normal oxygen concentration conditions. The aims of the current research were (1) to comprehensively identify and quantify the lipid composition of chicken embryonic brain under hypoxic and normoxic conditions, (2) to screen SDLs via multi-dimensional statistical analysis models, and (3) to elucidate the correlation and potential metabolic pathways of SDLs. The results provide a foundation for revealing dynamic lipid alterations, the metabolic pathways of differentially abundant lipids, and the screening of candidate hypoxia-responsive lipids in chicken embryo brains.

## 2. Materials and Methods

### 2.1. Ethics Statement

All animal procedures were performed according to the National Standards of the People’s Republic of China for Laboratory Animal Welfare and were approved by the Animal Care and Use Committee (ACUC) of Nanchang Normal University (No. 202409).

### 2.2. Chemicals and Reagents

The Chemicals and reagents used in this study were as follows: MS-grade methanol, MS-grade water, MS-grade acetonitrile, and HPLC-grade 2-propanol were purchased from Thermo Fisher (Waltham, MA, USA). GC-grade dichloromethane and HPLC-grade ammonium acetate were purchased from Sigma-Aldrich (St. Louis, MO, USA). GC-grade MTBE was purchased from Aladdin (Shanghai, China).

### 2.3. Brain Sample Collection

The fertilized eggs of TBCs (a high-altitude chicken breed) and DLCs (a lowland chicken breed) from the Experimental Chicken Farm at the China Agricultural University were collected and randomly transferred to normoxia (21% O_2_) and hypoxia (13% O_2_, which corresponds to the altitude of approximately 4000 m above sea level) incubators with a temperature of 37.8 °C and a relative humidity of 60%, respectively. Two independent incubators were applied for each oxygen level. Eggs were weighed and randomized across incubators to balance egg weight within each breed. Each incubator was considered an independent experimental unit for oxygen exposure. At embryonic day 18, six brain samples per group (HTBC18, NTBC18, HDLC18 and NDLC18) were harvested as previously described [18], with three embryos sampled from each of the two incubators within the same oxygen treatment to avoid pseudoreplication. Embryonic sex was not controlled during sampling and represents a potential source of biological variation. All brain tissues were immediately frozen in liquid nitrogen and stored at −80 °C until analysis.

### 2.4. Lipid Extraction

Lipids were isolated using a modified methyl tert-butyl ether (MTBE) extraction protocol. In brief, each sample was combined with 200 µL methanol, followed by the addition of 10 µL lipid internal standard mixture and 800 µL MTBE. The resultant suspension was thoroughly vortexed, subjected to 20 min of sonication at 4 °C, and then held at ambient temperature for 30 min. Phase separation was triggered by adding 200 µL MS-grade water; after vortex mixing, the mixture was centrifuged at 14,000 rpm for 15 min at 4 °C. The upper organic phase was harvested and evaporated to dryness under a gentle nitrogen stream. Prior to LC–MS measurement, dried lipid extracts were reconstituted in 200 µL of isopropanol/acetonitrile (9:1, *v*/*v*). The reconstituted solution was centrifuged again at 14,000× *g* and 4 °C for 15 min, and the resulting supernatant was loaded for instrumental detection.

### 2.5. LC-MS/MS Method for Lipid Analysis

Lipid profiling was implemented on an ultra-high-performance liquid chromatography platform (LC-30AD, Shimadzu Corporation, Kyoto, Japan) interfaced with a QTRAP 6500+ mass spectrometer (Sciex, Framingham, MA, USA). Two orthogonal separation columns were deployed for lipid fractionation: a Luna NH_2_ HILIC column (2.0 mm × 100 mm, 3 μm; Phenomenex, Torrance, CA, USA) and a Kinetex C18 reversed-phase column (2.1 mm × 100 mm, 2.6 μm; Phenomenex, Torrance, CA, USA).

For reversed-phase C18 separation, the column oven was maintained at 45 °C. Mobile phase A consisted of 70% acetonitrile and 30% ultrapure water supplemented with 5 mM ammonium acetate, whereas mobile phase B was pure isopropanol. The elution gradient ran at a flow rate of 0.35 mL/min: 20% B from 0 min, linearly increased to 60% B at 5 min, reached 100% B at 13 min, and returned to 20% B for re-equilibration between 13.1 and 17 min. The sample manager compartment was held at 10 °C throughout the entire analytical run.

For HILIC separation on the amino-bonded stationary phase, the column temperature was stabilized at 40 °C. Mobile phase A contained 2 mM ammonium acetate dissolved in a methanol–acetonitrile mixture (50:50, *v*/*v*), and mobile phase B was prepared with 2 mM ammonium acetate in acetonitrile–water (50:50, *v*/*v*). Elution was conducted at a flow rate of 0.40 mL/min with the following gradient timetable: isocratic 3% B for 0–3 min, linear ramp from 3% to 100% B over 3–13 min, isocratic 100% B for 13–17 min, and re-equilibration at 3% B from 17.1 to 22 min.

The 6500+ QTRAP mass spectrometer was operated with fast polarity switching between positive and negative electrospray ionization modes. Optimized ESI(+) source parameters were set as follows: source temperature of 400 °C; ion source gas 1 (GS1) = 50 psi; ion source gas 2 (GS2) = 55 psi; curtain gas (CUR) = 35 psi; ion spray voltage (IS) = +3000 V. For ESI(−) acquisition, the source temperature, GS1, GS2 and CUR were kept identical, while the ion spray voltage was adjusted to −2500 V. Multiple reaction monitoring (MRM) was adopted to acquire lipid data. Pooled quality control (QC) specimens were interspersed among sample injections to monitor the stability and repeatability of the whole analytical workflow.

Lipid quantification was conducted using a proprietary LC–MRM-MS lipidomic assay from APTBIO. The full MRM transition list is commercially confidential and unavailable. Lipid annotations were assigned according to Metabolomics Standards Initiative (MSI) confidence criteria, supported by class-specific diagnostic fragmentation, chromatographic retention time and isotope internal standard matching.

### 2.6. Data Processing

Raw data were processed using Sciex OS software (version 3.1.6). Peak integration was carried out on MRM chromatograms to compute the peak-area ratio between each target lipid analyte and its corresponding internal standard, which was subsequently applied for relative content calculation. All pooled QC specimens were processed alongside experimental biological samples. Lipid features detected in QC samples with a coefficient of variation (CV) below 30% were retained as reproducible analytical measurements.

### 2.7. Statistical Analysis

Lipid data were log_2_-transformed before statistical analysis to reduce skewed distribution. Levene’s test was performed to assess homogeneity of variances. A two-way ANOVA was applied to evaluate the main effects of breed (TBC vs. DLC), oxygen condition (hypoxia vs. normoxia), and their breed × oxygen interaction on lipid species and lipid subclass levels across four groups (NTBC18, HTBC18, NDLC18, HDLC18). When the breed × oxygen interaction was statistically significant, simple-effect analysis was conducted to perform pairwise comparisons within each breed: HTBC18 vs. NTBC18 and HDLC18 vs. NDLC18. To account for the incubator-level clustering of embryos, incubator identity was included as a covariate in the statistical models, together with fixed factors of breed (TBC vs. DLC) and oxygen treatment (normoxia vs. hypoxia). Owing to only two incubator replicates per oxygen condition, fitting incubator as a random effect was not statistically feasible, as random-effects variance estimates tend to be unreliable with just two groups. This experimental constraint is acknowledged as a limitation of the study. Data analysis and graph drawing were performed in GraphPad Prism Version 9.5 (San Diego, CA, USA). Multivariate statistical analyses, including PCA, PLS-DA and OPLS-DA, were applied to the lipidomics data. For supervised PLS-DA and OPLS-DA modeling, 7-fold cross-validation and permutation testing (*n* = 200 permutations) were performed to assess model performance. The statisticsR^2^X, R^2^Y, and Q^2^ were calculated to describe the explained variation and predictive ability. It should be noted that cross-validation was implemented at the individual embryo level. Considering that oxygen exposure was applied at the incubator level in our experimental design, these supervised models serve only as exploratory tools to visualize lipidomic shifts, rather than robust proof of biological discrimination between groups. The screening criteria for the SDLs were fold change in the expression level (FC) > 1.5 or FC < 0.67, FDR-adjusted q-value < 0.05 and the VIP value > 1. KEGG pathway enrichment of SDLs was performed using over-representation analysis (ORA). The background set consisted of all lipids detected. SDLs were mapped to KEGG pathways based on matched KEGG compound IDs. Fisher’s exact test was used to compute nominal *p* values, and the Benjamini–Hochberg method was used to control the false discovery rate, yielding FDR adjusted q-values. For each enriched pathway, the number of mapped SDLs, FDR q-value were calculated and reported.

## 3. Results

### 3.1. Reliability of Analytical Methods

The characteristics of metabolic profiles from lipid extracts and QC samples were investigated to assess the reproducibility of the sample preparation procedure and the reliability of analytical methods. Four identical QC samples were made by mixing aliquots of lipid extracts from 6 NTBC18, 6 HTBC18, 6NDLC18 and 6 HDLC18 samples. Firstly, the retention time and peak area of total ion chromatogram of the QC samples were compared by spectral overlap (Figure S1A,B), and overlapped well, indicating that excellent stability of the instrument. Subsequently, to more intuitively evaluate the repeatability of the samples, the Pearson correlation analysis among QC samples showed that the correlation coefficients were all above 0.99 (Figure S2A). PCA of the overall samples indicated that QC samples were closely clustered together and located in the middle of the other samples on PC1 (Figure S2B). Hotelling’s T2 test of the overall samples suggested that QC samples were within the 99% confidence interval (Figure S2C). These results implied that the within-group repeatability of QC samples was highly excellent. Furthermore, further analysis revealed that the fluctuations of the QC samples multivariate control chart (MCC) were within two standard deviations (Figure S3A) and the proportion of peaks with relative standard deviation (RSD) ≤ 30% among all the peaks was over 80% in the QC samples (Figure S3B), which demonstrated that the data quality were reliable. Therefore, the results showed the reliability of the analytical methods employed in this study.

### 3.2. Lipid Profiles of TBC and DLC Samples

**Figure 1:**
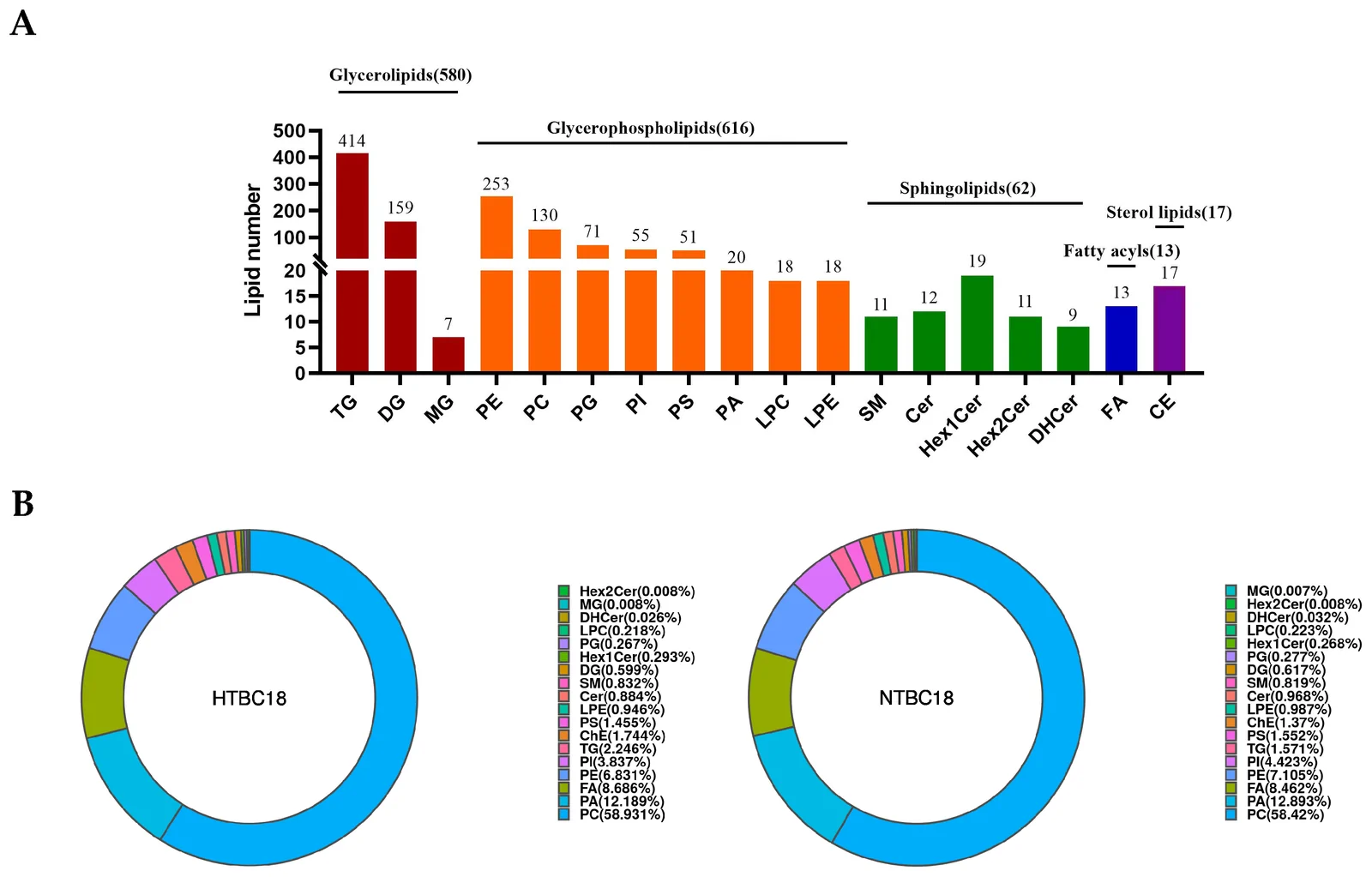

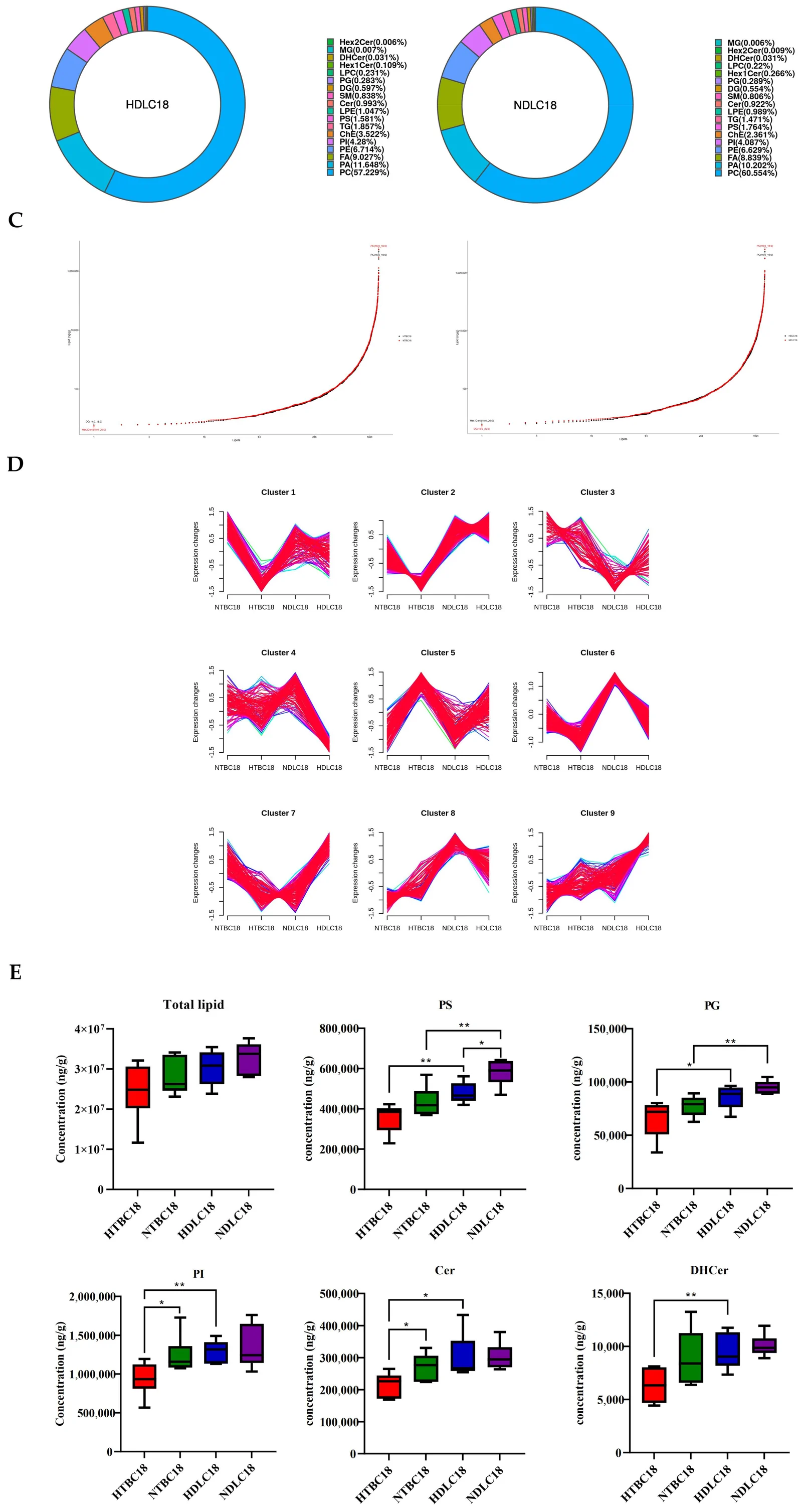
Lipid composition identified in the embryonic brain tissue of chickens. (**A**) Lipid subclasses and the number of lipid species. The x−axis represents the detected lipid subclasses. The y−axis indicates the number of lipid species within each subclass. (**B**) Lipid subclasses composition in each group. Different lipid subclasses are distinguished by different colors, and the area of each color block represents its proportion. (**C**) The dynamic distribution of lipid species content in each group. Each dot represents a lipid molecule, which are sorted by content from low to high. The y−axis shows the absolute content of each lipid species. The names of the lipid species with the lowest and highest content are labeled at the lower-left and upper-right corners of the plot, respectively. Different groups are distinguished by distinct colors. (**D**) Variation trends of identified lipid species across sample groups. The x−axis represents different groups, and the y−axis indicates the changes in normalized expression levels. Each line within a cluster corresponds to a metabolite with a shared expression pattern. (**E**) The comparison of total lipids and lipid subclasses between TBCs and DLCs under hypoxia and normoxia. * means q < 0.05, ** means q < 0.01.

A total of 1288 lipid species from TBC18 and DLC18 samples were identified, as shown in Figure 1A (Table S1), which were grouped into 18 lipid subclasses, including 13 FAs, 414 TGs, 159 DGs, 7 MGs, 253 PEs, 130 PCs, 71 PGs, 51 PSs, 55PIs, 20 PAs, 18 LPCs, 18 LPEs, 11 SMs, 12 Cers, 9 DHCers, 19 Hex1Cers, 11 Hex2Cers and 17 CEs. Among them, TGs were the most abundant lipid, accounting for 32.14% of the total number of lipids, followed by PEs (19.64%), DGs (12.34%) and PCs (10.09%), which determined the abundance of lipid species in the chicken embryo brain. All the lipids can be further classified into 5 classes, namely 580 GLs (45.03%), 616 GPs (47.83%), 62 SPs (4.81%), 13 FAs (1.01%) and 17 STs (1.32%). Obviously, GPs were the most abundant lipid class, followed by GLs, which together accounted for 92.86% of the total lipids classes. Moreover, the sum of all lipids within the same subclass was used to calculate the relative content of each lipid subclass. As revealed in Figure 1B, PCs was the lipid subclass with the highest percentage of abundance in HTBC18 (58.931%), NTBC18 (58.420%), HDLC18 (57.229%) and NDLC18 (60.554%), followed by PAs (HTBC18 12.189%, NTBC18 12.893%, HDLC18 11.648% and NDLC18 10.202%), FAs (HTBC18 8.686%, NTBC18 8.462%, HDLC18 9.027% and NDLC18 8.839%) and PEs (HTBC18 6.714%, NTBC18 7.105%, HDLC18 6.714% and NDLC18 6.629%). PC (16:0_16:0) was the lipid species with the highest content in each group, whereas lipid species with the lowest content included DG (14:0_18:3) in the HTBC18, Hex2Cer (d18:0_20:0) in the NTBC18 group, Hex1Cer (d18:1_26:0) in the HDLC18 group and DG (15:0_20:3) in the NDLC18 group (Figure 1C). As illustrated in Figure 1D, the expression trends of all lipid species were divided into 9 different expression modules. At the same time, the contents of total lipids and lipid subclasses were compared in each group (HTBC18, NTBC18, HDLC18 and NDLC18). No differences in total lipids were observed, and the contents of PSs, PGs, PIs, Cers and DHCers were significantly different between TBCs and DLCs under hypoxia or normoxia (Figure 1E). Interestingly, the lipid subclasses with significant differences all had the lowest contents in the HTBC18 group (Figure 1E).

### 3.3. Identification of SDLs Between TBC and DLC Samples

**Figure 2:**
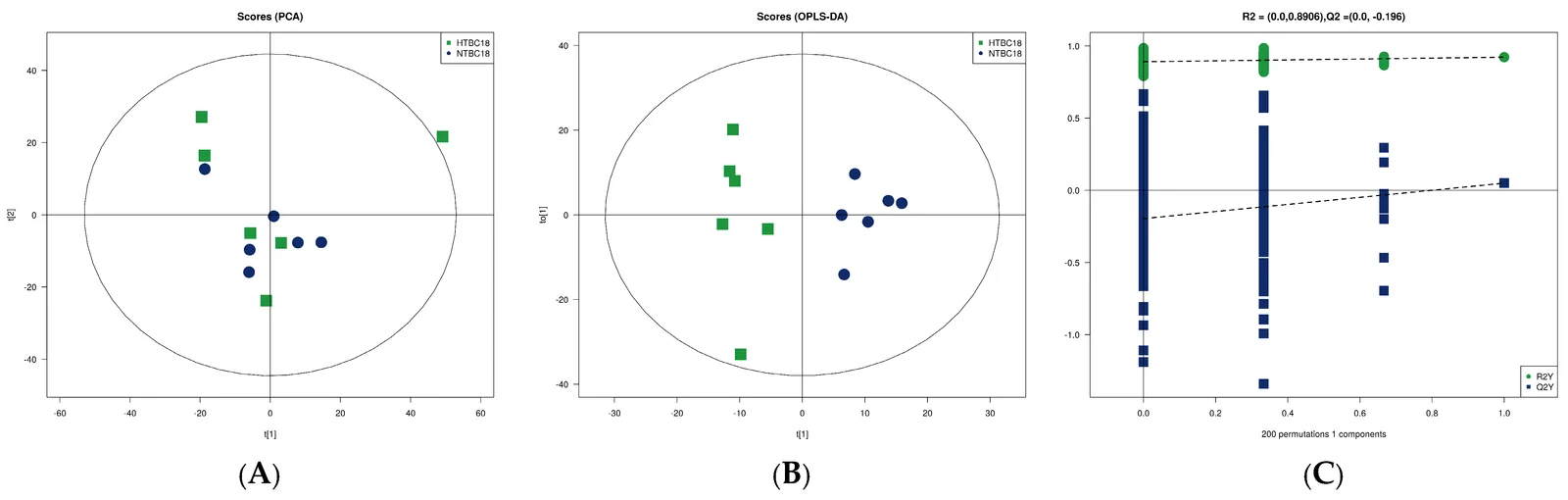

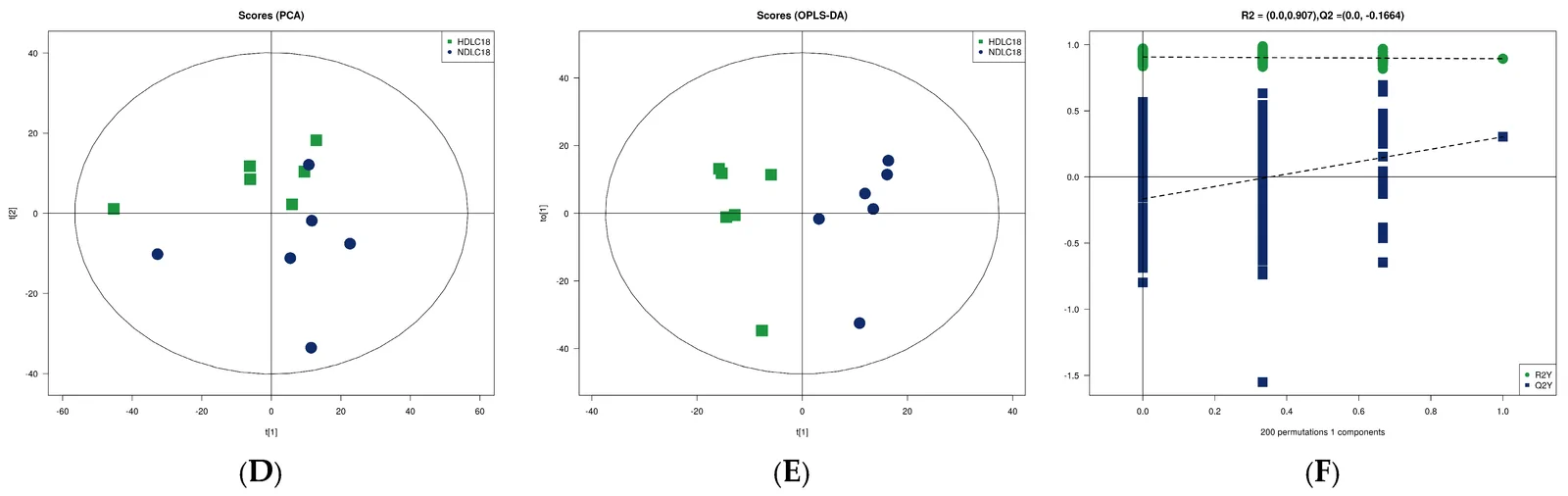
Multivariate analysis of lipidomics in TBCs and DLCs. (**A**) PCA score plot between HTBC18 and NTBC18. (**B**,**C**) OPLS−DA score plot and permutation test between HTBC18 and NTBC18. (**D**) PCA score plot between HDLC18 and NDLC18. (**E**,**F**) OPLS−DA score plot and permutation test between HDLC18 and NDLC18.

In order to deeply explore the overall distribution of lipids in chicken embryo brains under different oxygen concentrations and to screen the different changes of lipid metabolites under hypoxia, we executed multivariate statistical analysis and univariate statistical analysis, including PCA, PLS-DA and OPLS-DA. The unsupervised PCA plot was used to visualize the overall distribution of lipids and differences between samples from different groups. The R2X parameter values of the PCA model obtained through 7-fold cross-validation are 0.606 (HTBC18 vs. NTBC18) and 0.601 (HDLC18 vs. NDLC18) (Figure 2A,D). However, there were overlaps in both comparison groups, indicating that there was no clear separation among the groups. Supervised PLS-DA was employed to reveal the separation of samples from different groups. As illustrated in the PLS-DA score plots, clear separation was observed between the groups (Figure S4A,C). The PLS-DA model was tested using the permutation test to ensure its validity. The parameter R2 values (0.0, 0.889) and Q2 values (0.0, −0.1902) in the HTBC18 vs. NTBC18 (Figure S4B). For the HDLC18 vs. NDLC18, R2 values (0.0, 0.9825) and Q2 values (0.0, 0.1933) (Figure S4D), indicating that the model fit data well. Compared with the PLS-DA model, the OPLS-DA model can filter out noise unrelated to the classification information, enhancing the analytical ability and effectiveness of the model. The OPLS-DA score plot also shows the clear separation of each group (Figure 2B,E). The prediction accuracy and goodness of fit of the OPLS-DA model were evaluated through 7-fold cross-validation and 200-permutation test. As summarized in Figure 2C,F, in the HTBC18 vs. NTBC18 comparison, the permutation test yielded R2 (0.0, 0.8906) and Q2 values (0.0, −0.196) (Figure 2C). The established OPLS-DA model had R^2^X = 0.291, R^2^Y = 0.922, and Q^2^ = 0.522. For the HDLC18 vs. NDLC18 comparison, R2 values (0.0, 0.907) and Q2 values (0.0, −0.1644) (Figure 2F), and OPLS-DA model parameters R^2^X = 0.519, R^2^Y = 0.989, and Q^2^ = 0.545. Permutation testing and 7-fold cross-validation indicated no obvious signs of overfitting for the constructed models. Nevertheless, given the limited sample size and incubator-based treatment structure, these supervised discrimination results should be interpreted with caution and considered exploratory.

**Figure 3:**
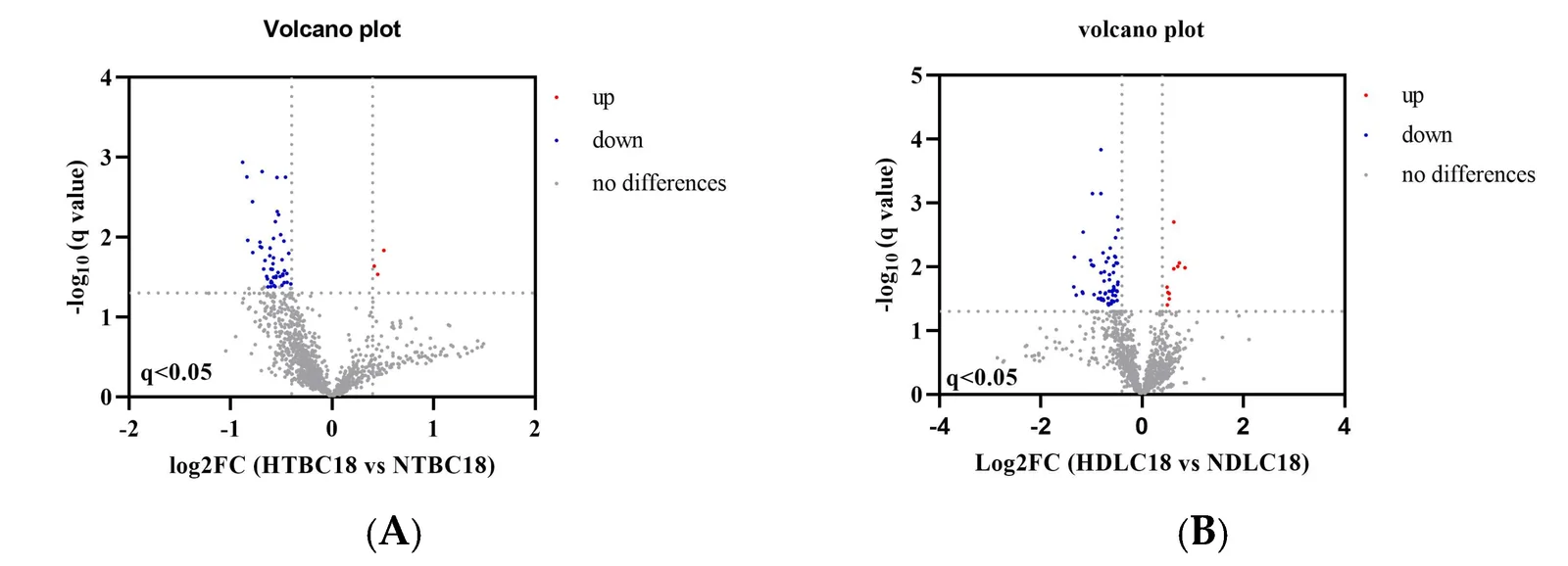

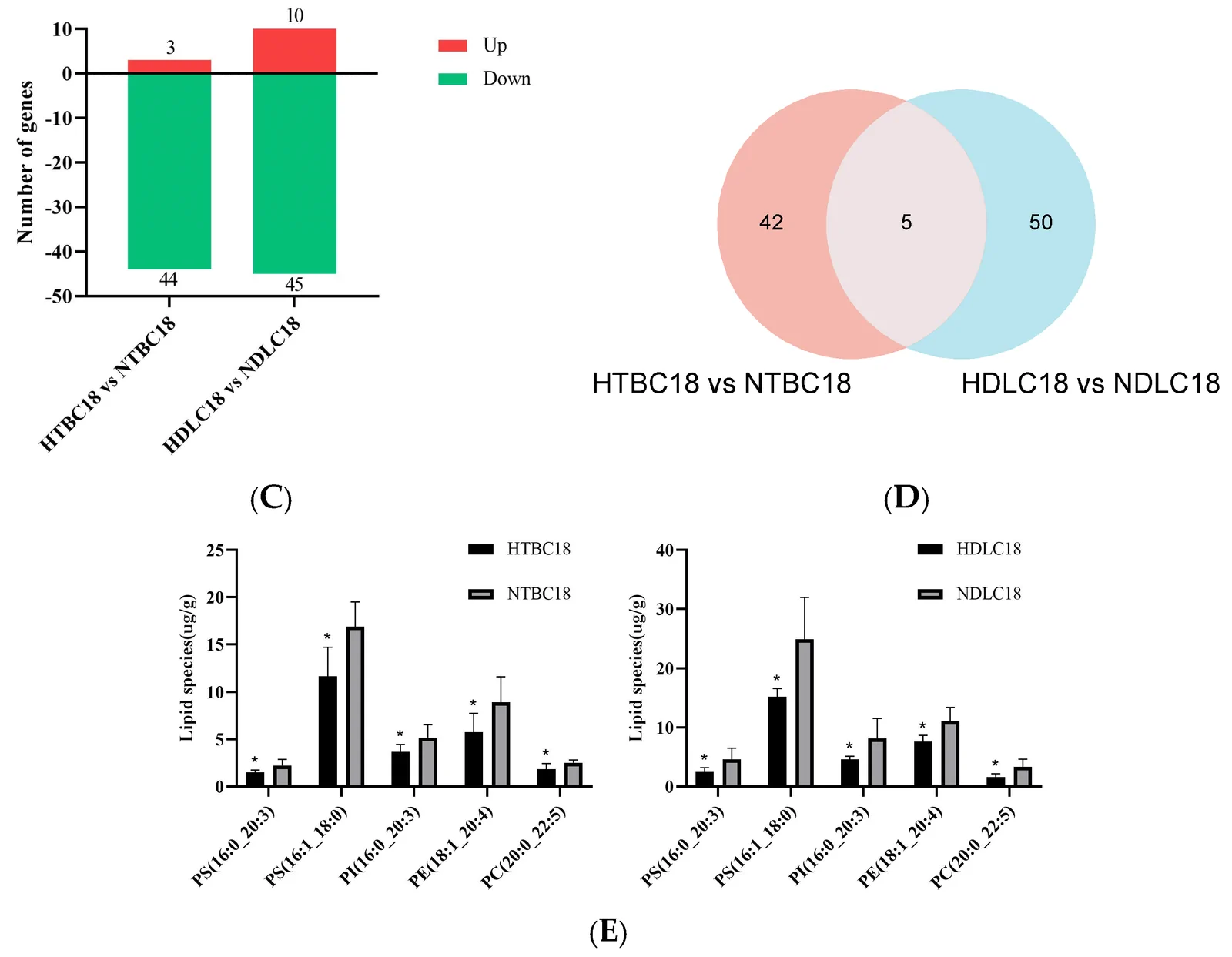
The SDLs between TBCs and DLCs. The volcano plot (FC > 1.5 or FC < 0.67, q < 0.05) for HTBC18 vs. NTBC18 (**A**) and HDLC18 vs. NDLC18 (**B**). (**C**) The final SDLs (VIP > 1, q < 0.05) of HTBC18 vs. NTBC18 and HDLC18 vs. NDLC18. (**D**) The Venn diagram of the shared SDLs between HTBC18 vs. NTBC18 and HDLC18 vs. NDLC18. (**E**) Five shared lipid species in TBCs and DLCs under normoxia and hypoxia. * q < 0.05.

In this study, lipid species that were significant differences abundant between groups are shown in volcano plots with FC > 1.5 or FC < 0.67 and q < 0.05. A total of 52 SDLs (3 up-regulated, 49 down-regulated) in the HTBC18 and NTBC18 groups (Figure 3A), whereas 72 SDLs (10 up, 62 down) in the HDLC18 and NDLC18 groups (Figure 3B). Furthermore, the screening criteria for SDLs between groups were based on the variable weight values of the OPLS-DA model (VIP > 1) and q < 0.05. A total of 47 SDLs (3 up-regulated and 44 down-regulated) were identified between the HTBC18 and NTBC18 groups (Figure 3C), 55 SDLs (10 up-regulated and 45 down-regulated) were identified in the HDLC18 compared to NDLC18 (Figure 3C). Only 5 SDLs were shared between HTBC18 vs. NTBC18 and HDLC18 vs. NDLC18 (Figure 3D), among which PS (16:0_20:3) was differentially between TBCs and DLCs regardless of normoxicity or hypoxia, PS (16:1_18:0) was only differentially between NTBC18 vs. NDLC18, and the other 3 SDLs (PI (16:0_20:3), PE (18:1_20:4) and PC (20:0_22:5)) showed no differences among chicken breeds (Figure 3E).

### 3.4. Correlation Analysis of SDL TBC and DLC Samples

**Figure 4:**
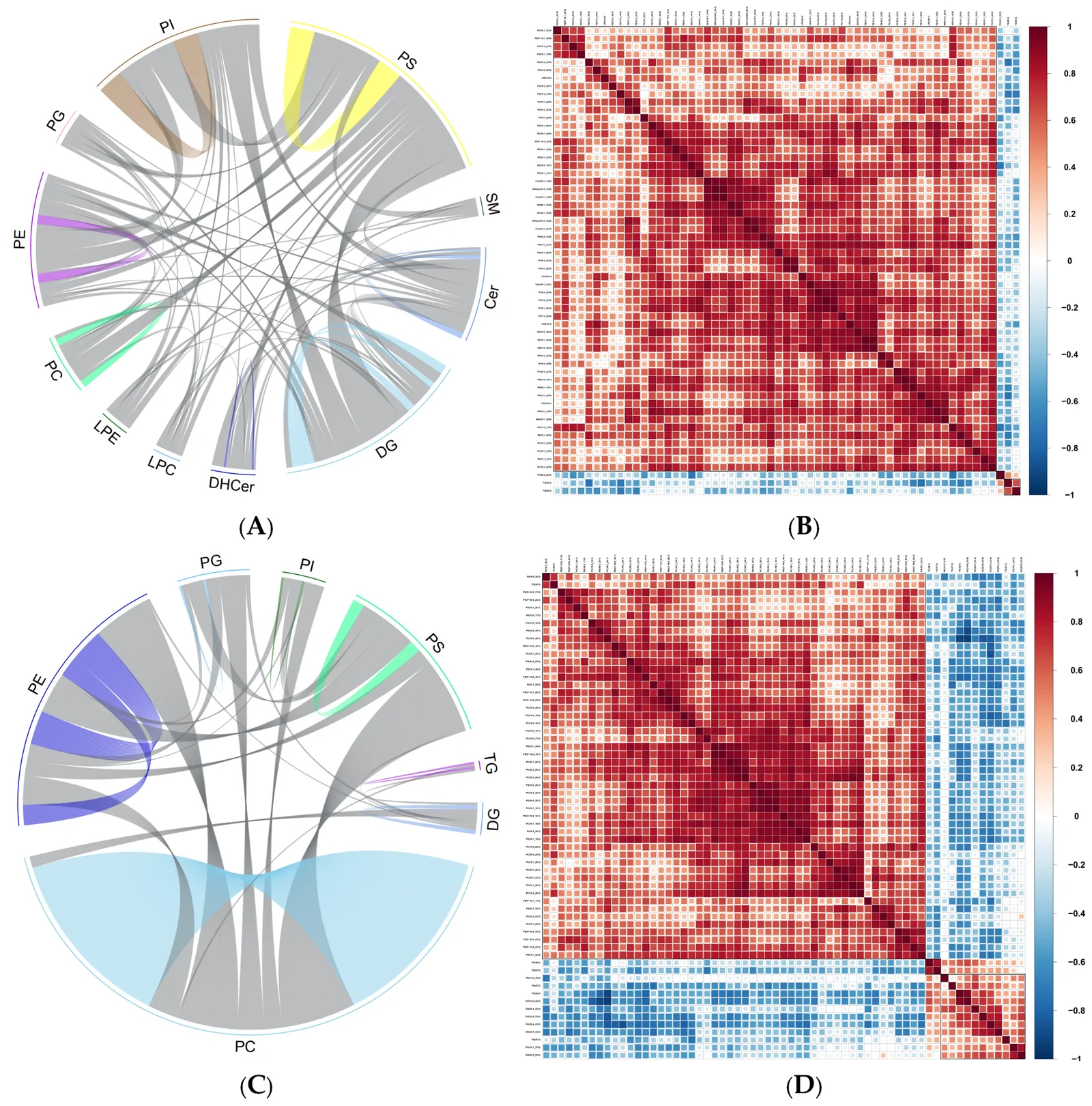
The co−regulatory relationships of SDLs between HTBC18 vs. NTBC18 (**A**,**B**) and HDLC18 vs. NDLC18 (**C**,**D**). (**A**,**C**) The chord diagram of the co−regulation relationship of different lipids (|r| > 0.8 and *p* value < 0.05). Inner nodes: differential lipids; outer arcs: lipid subclasses; colored lines: intra-subclass correlation; dark gray lines: inter-subclass correlation. (**B**,**D**) Correlation cluster heatmap of different lipids (VIP > 1, *p* value < 0.05). Red: positive correlation; blue: negative correlation; darker color: stronger correlation.

To better understand the degree of metabolic proximities and mutual regulation relationship between lipids, a correlation analysis was conducted on the SDLs. As shown in Figure 4, PSs, DGs, PIs and PEs had the highest levels of interaction with other lipid species in the HTBC18 vs. NTBC18 groups (Figure 4A). In contrast, in the HDLC18 vs. NDLC18 groups, PCs, PEs and PSs demonstrated more interactions with other lipid species other lipids (Figure 4B). PS (18:1_ 22:4) and DG (16:0_20:3), PS (18:1_22:4) and PI (18:1_20:4), PS (18:1_22:4) and PE (18:1_20:5) had more connections and a higher correlation to other species in the HTBC18 vs. NTBC18 groups than in the HDLC18 vs. NDLC18 groups (Figure 4C). In contrast, the HDLC18 vs. NDLC18 groups than the HTBC18 vs. NTBC18 groups, PC (16:0_20:3) and PE (14:0_16:1), PC (20:3_20:3) and PS (16:0_20:3), PE (0_16:0_16:1) and PS (16:0_20:3) had more connections and a higher correlation to other species (Figure 4D). Lipid saturation was further analyzed in PS, DG, and PI between HTBC18 and NTBC18 (Figure S5A–C), and PC, PE, and PS between HDLC18 and NDLC18 (Figure S5D–F). Lipid species with the same unsaturated bond but different contents have lower levels in hypoxic conditions compared to normoxic conditions.

### 3.5. KEGG Pathway Analysis of SDLs

**Figure 5:**
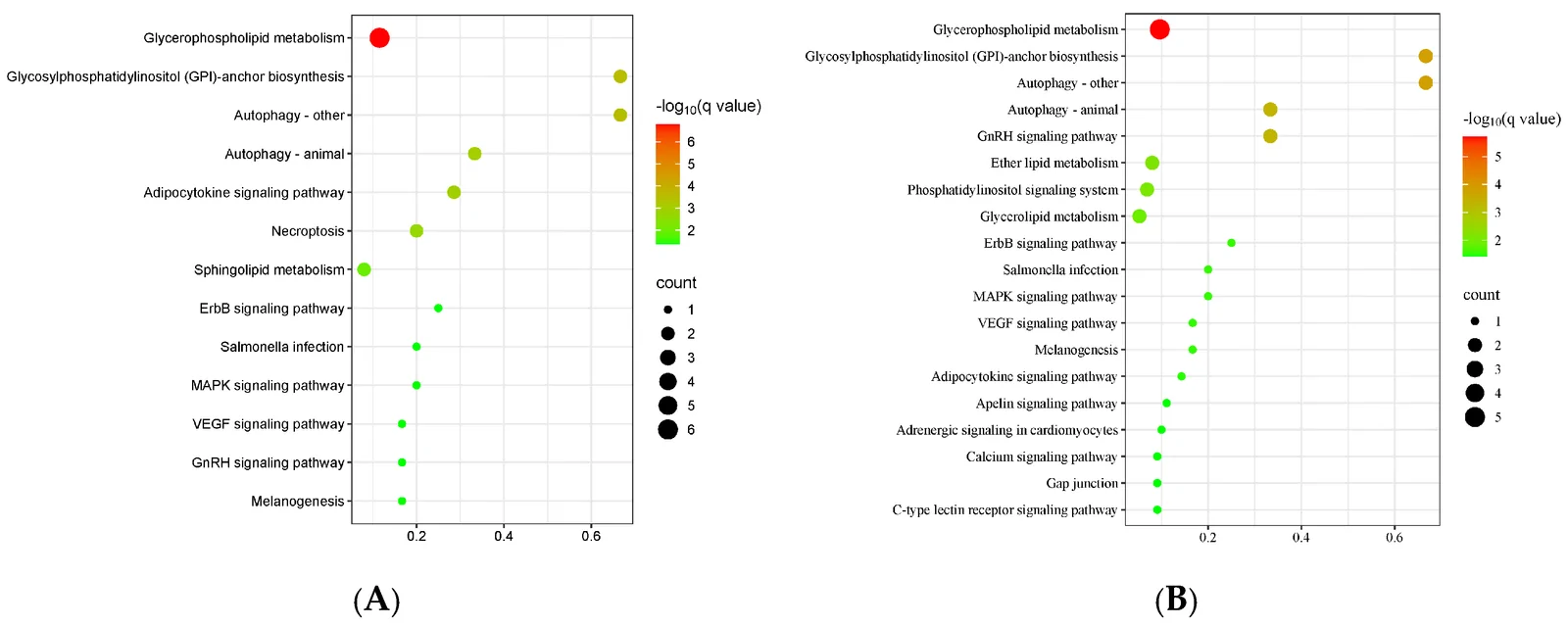
Analysis of KEGG pathways for the SDLs in HTBC18 vs. NTBC18 (**A**) and HDLC18 vs. NDLC18 (**B**).

To investigate whether the metabolism of chicken embryonic brain lipids (TBCs and DLCs) is affected by hypoxia, 47 and 55 SDLs were mapped to the KEGG pathway database, and finally, among which 13 (Table S2) and 19 (Table S3) KEGG pathways were significantly enriched, respectively (Figure 5A,B). Among these significantly enriched pathways, regardless of whether TBCs or DLCs, GPs’ metabolism was the most significantly relevant metabolic pathway, followed by glycosylphosphatidylinositol (GPI)-anchor biosynthesis and autophagy. Interestingly, all 5 shared SDLs were involved in the GPs’ metabolism. These lipids were determined as potential hypoxia-responsive lipids, but further experiments will be carried out for verification.

## 4. Discussion

The brain is the second most lipid-rich organ in the body, with lipid content only lower than that of adipose tissue [36]. Brain lipids are mainly composed of cholesterol, phospholipids (such as PCs, PEs), and SPs, which play essential roles during embryonic brain development [36]. Cholesterols are mostly generated by astrocytes and transported to neurons through the formation of cholesterol-rich lipoproteins, used for neurite maintenance and synaptic connectivity [36,37]. GPs, including PCs and PEs, are essential phospholipid components of cell membranes, and alterations in their composition can impair the stability, permeability and fluidity of neural membranes, thereby triggering neurological disorders [36,38]. SPs, usually observed in the nervous system, are the main components of cellular membranes, and are involved in brain function regulation, especially neurogenesis and synaptogenesis [36,39]. The brain is highly sensitive to alterations in oxygen concentration. Hypoxic stress not only affects cerebral energy metabolism but also leads to alterations in lipid metabolism [24]. Hence, comprehensive brain lipidomic analysis is of great value for uncovering the molecular mechanisms of hypoxia adaptation. Nevertheless, systematic investigations on the brain lipidome under hypoxic conditions remain insufficient. In this study, we employed an UPHLC-Qtrap MS-based targeted lipidomic technique to characterize dynamic molecular features of the lipidome in day 18 chicken embryonic brains under hypoxia and screen potential hypoxia-responsive lipids. A total of 1288 lipid species belonging to 18 lipid subclasses were characterized in both TBC and DLC groups (Figure 1A), which substantially improves our understanding of lipid composition in chicken embryonic brains. Lipid class composition analysis revealed that PCs, PAs, FAs, PEs, and PIs were the top five most abundant lipid subclasses across the four groups (HTBC18, NTBC18, HDLC18, and NDLC18, Figure 1B). The results indicated that the brain of chicken embryos is rich in GPs. However, hypoxia exerted no significant effect on total lipid content in TBC and DLC (Figure 1D), which was consistent with previous findings from untargeted lipidomic analysis of chicken embryo brain [20]. Notably, the significantly altered lipid subclasses, including PSs, PGs, PIs, Cers and DHCers, all exhibited the lowest abundance in the HTBC18 group. Previous hypoxia experiments in rat models have reported decreased levels of PSs and PIs in the brain [36,40], which supports our present results.

Based on the changes of brain lipid molecules in chicken embryos incubated under normoxic and hypoxic conditions, we identified 47 SDLs between HTBC18 and NTBC18, and 55 SDLs in the HDLC18 vs. NDLC18, among which only five lipid species, PI (16:0_20:3), PE (18:1_20:4), PC (20:0_22:5), PS (16:1_18:0), and PS (16:0_20:3), were shared (Figure 3C–E). This indicates that embryonic brains of high- and low-altitude chickens exhibit divergent lipid response patterns to hypoxic stress. Accumulating evidence from human high-altitude exposure investigations and mammalian cerebral hypoxia models has confirmed that hypoxic stress markedly disturbs the metabolism of GPs, SPs, and lipid droplets in the central nervous system, and lipid metabolism serves as a core metabolic signature in response to high-altitude hypoxia [21,22,24,41,]. In the present study, five shared hypoxia-responsive lipid molecules identified in both TBCs and DLCs belonged to the GPs family. As essential functional components responsible for maintaining neural membrane integrity, regulating membrane fluidity, and mediating lipid signal transduction, five lipid species exhibited consistent hypoxic responses in embryonic brains of both TBCs and DLCs, demonstrating that GPs were associated with the brain lipidomic response to hypoxia in chicken embryos. Therefore, these five lipid molecules can be regarded as candidate hypoxia-responsive lipids in chicken embryonic brains. Furthermore, PS (16:0_20:3) exhibited differential abundant between TBC and DLC under both normoxic and hypoxic conditions, whereas PS (16:1_18:0) was differentially only between NTBC18 and NDLC18. As major acidic phospholipids in the brain, PSs are closely associated with neuronal survival, synaptic outgrowth and synaptogenesis [20]. Neurons can synthesize PSs using L threonine derived from astrocyte produced glucose, and hypoxia can trigger the translocation of PSs [20]. The differences in PSs between TBCs and DLCs suggested that hypoxia-associated PSs’ metabolism may be correlated with processes underlying embryonic neural development. However, an important limitation of this study is the limited number of incubator replicates (n = 2 for each oxygen condition). Since oxygen exposure was applied at the incubator level, we cannot completely disentangle incubator-specific variation from hypoxia-induced lipidomic changes, even after statistical adjustment for incubator identity. This experimental constraint should be considered when interpreting all lipidomic results. Future work with a greater number of independent incubator replicates is needed to better partition incubator noise and validate the hypoxia-responsive lipid changes observed here.

Further metabolic pathway enrichment analysis of SDLs identified a total of 13 and 19 lipid metabolic pathways. Among them, the GPs’ metabolism pathway exhibited the most significant enrichment of SDLs, followed by the GPI anchor biosynthesis and autophagy. The results confirmed that GPs’ metabolism was strongly associated with the brain lipidomic response to hypoxic stress in chicken embryonic, and this response pattern is highly consistent with the lipid response characteristics of the central nervous system under hypoxic conditions in mammals [24]. Hypoxic stress significantly interferes with the synthesis, acyl chain modification, hydrolysis and transport of GPs, disrupts neural lipid metabolism homeostasis, and further induces cell membrane structural damage and neurodevelopmental abnormalities [24]. Based on our results, it is speculated that hypoxia triggers alterations in GPs’ metabolism in the embryonic chicken brain, which may represent one of the measurable lipid changes accompanying hypoxic challenge. In conclusion, the current results reveal an association between hypoxia exposure and GPs’ abundance in the brain of chicken embryos.

## 5. Conclusions

Herein, a targeted lipidomics strategy using isotope internal standard combined with UPHLC-Qtrap MS was used to explore the brain lipid profiles of day 18 embryos from TBCs and DLCs under hypoxic and normoxic conditions. Overall, 1288 lipid species belonging to 18 lipid subclasses were identified; 47 SDLs were screened in the HTBC18 vs. NTBC18 groups, and 55 SDLs were detected in the HDLC18 vs. NDLC18 groups. Five shared lipid molecules (PI (16:0_20:3), PE (18:1_20:4), PC (20:0_22:5), PS (16:1_18:0), and PS (16:0_20:3)) were identified as candidate hypoxia-responsive lipids in chicken embryonic brain. Moreover, the lipid metabolism pathways closely related to SDLs were identified. Among them, glycerophospholipid metabolism was dominant, followed by glycosylphosphatidylinositol (GPI)-anchor biosynthesis and autophagy. The novelty of this work is the identification of conserved brain lipid signatures in two chicken lines differing in hypoxia tolerance, offering new lipid-level evidence for embryonic brain hypoxic adaptation in poultry. These findings support molecular breeding for hypoxia-resistant chickens. Future studies should validate these candidate hypoxia-responsive lipids in larger independent sample sets and combine multi-omics to uncover upstream regulatory mechanisms of brain lipid remodeling under hypoxia. However, this study has limitations. Lipid profiling was restricted to E18 brain tissue, which ignores potential stage-, tissue-, and sex-specific lipid responses. Further functional assays are necessary to establish causal relationships between these lipids and embryonic hypoxic adaptation.

## Data Availability

The original data presented in the study are included in the article. Further inquiries are available from the corresponding author upon reasonable request.

## Supplementary Materials

The following supporting information can be downloaded at: https://www.mdpi.com/article/10.3390/ani16213515/s1, Figure S1: The overlapping spectrum of the total ion current of QC samples. (A) C18 chromatographic column. (B) Amino chromatographic column. Horizontal axis: the retention time, Vertical axis: the intensity of the peak; Figure S2: The repeatability analysis of the sample. (A) The correlation map of QC samples. (B) PCA analysis of the overall sample, t[1] represents PC1, and t[2] represents PC2. (C) The Hotellings T2 plot of the overall sample; Figure S3: The reliability of data quality. The MCC diagram (A) and RSD (B) of QC samples; Figure S4: Multivariate analysis of lipidomics in TBCs and DLCs. PLS-DA score plot (A) and permutation test (B) between HTBC18 and NTBC18. PLS-DA score plot (C) and permutation test (D) between HDLC18 and NDLC18; Figure S5: Lipid chain length analysis in HTBC18 vs NTBC18 (A-C) and HDLC18 vs NDLC18 (D-F). Table S1: Detailed information of the 1288 identified lipid species; Table S2: The significantly enriched KEGG pathways for the SDLs in HTBC18 vs NTBC18; Table S3: The significantly enriched KEGG pathways for the SDLs in HDLC18 vs NDLC18.

## Abbreviations

The following abbreviations are used in this manuscript:

TBCs	Tibetan chickens
DLCs	Dwarf laying chickens
SDLs	Significantly differentially abundant lipids
LC-MS	Liquid chromatography-mass spectrometry
PCA	Principal component analysis
PLS-DA	Partial least squares discrimination analysis
OPLS-DA	Orthogonal partial least squares discrimination analysis
VIP	Variable importance in projection
FAs	Fatty acyls
TGs	Triglycerides
DGs	Diglycerides
MGs	Monoglycerides
PEs	Phosphatidylethanolamines
PCs	Phosphatidylcholines
PGs	Phosphatidylglycerols
PSs	Phosphatidylserines
PIs	Phosphatidylinositols
PAs	Phosphatidic acids
LPCs	Lysophosphatidylcholines
LPEs	Lysophosphatidylethanolamines
SMs	Sphingomyelines
Cers	Ceramides
DHCers	Dihydroceramides
Hex1Cers	Hexosylceramides
Hex2Cers	Dihexosylceramides
CEs	Cholesteryl esters
GLs	glycerolipids
GPs	glycerophospholipids
SPs	sphingolipids
STs	sterol lipids
