# Supplementary material for: Targeted Lipidomics Insights into Lipid Discrepancies in Chicken Embryonic Brains Under Hypoxia Based on UHPLC-QTRAP-MS
Source: Animals (Basel). 2026 Nov 6;16(21):3515. doi: 10.3390/ani16213515 (PMC13762403; doi:10.3390/ani16213515)
Supplement: Supplementary file 1 [file animals-16-03515-s001.zip › Supplementary figure.pdf]

A B

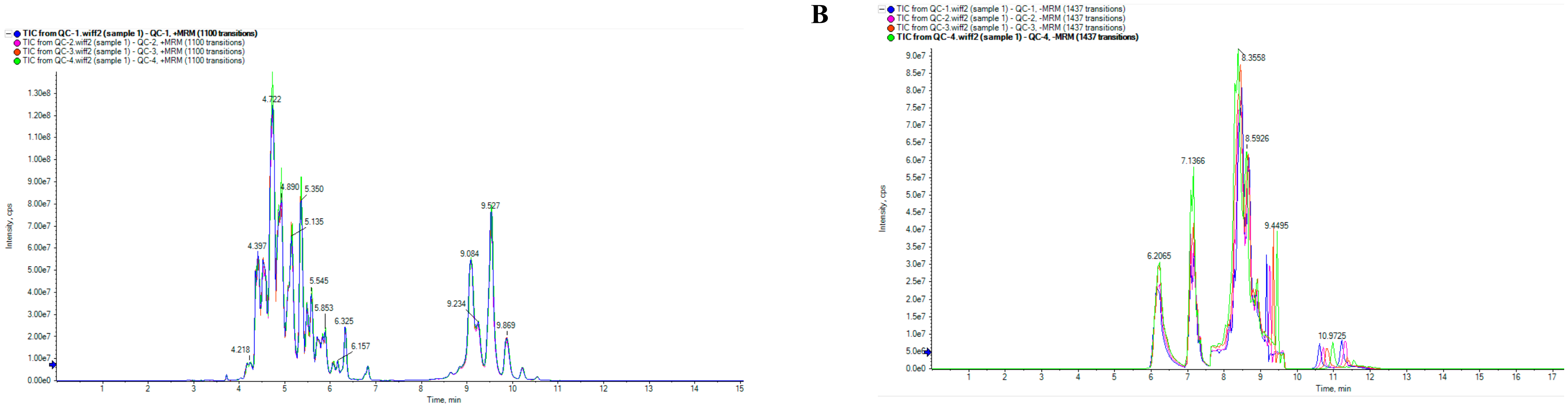

Figure S1: The overlapping spectrum of the total ion current of QC samples. (A) C18 chromatographic column. (B) Amino chromatographic column. Horizontal axis: the retention time, Vertical axis: the intensity of the peak.

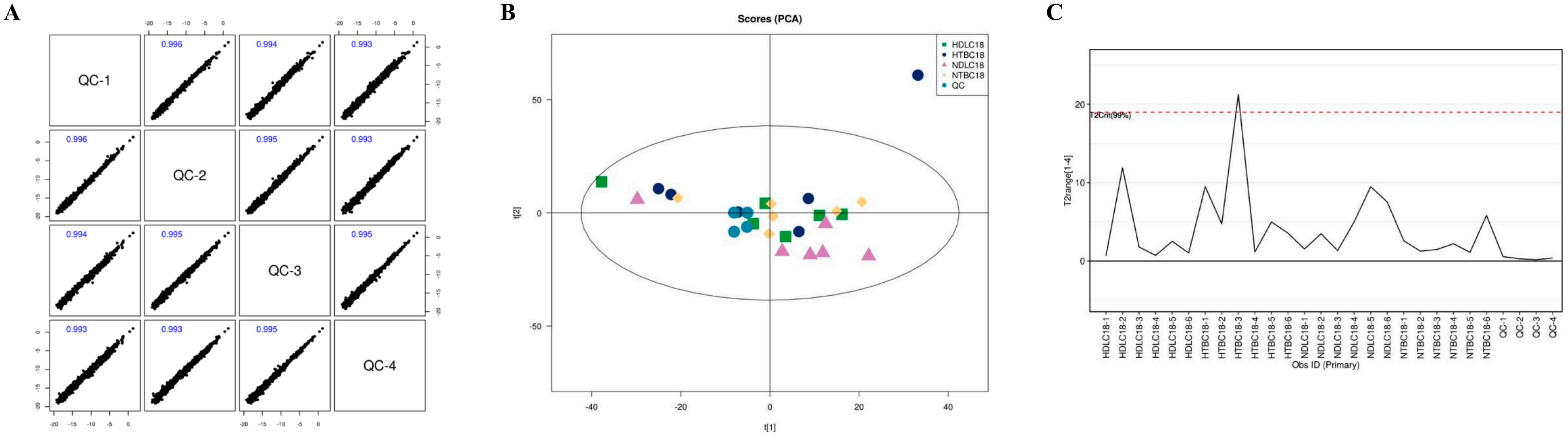

Figure S2: The repeatability analysis of the sample. (A) The correlation map of QC samples. (B) PCA analysis of the overall sample, t[1] represents PC1, and t[2] represents PC2. (C) The Hotellings T2 plot of the overall sample.

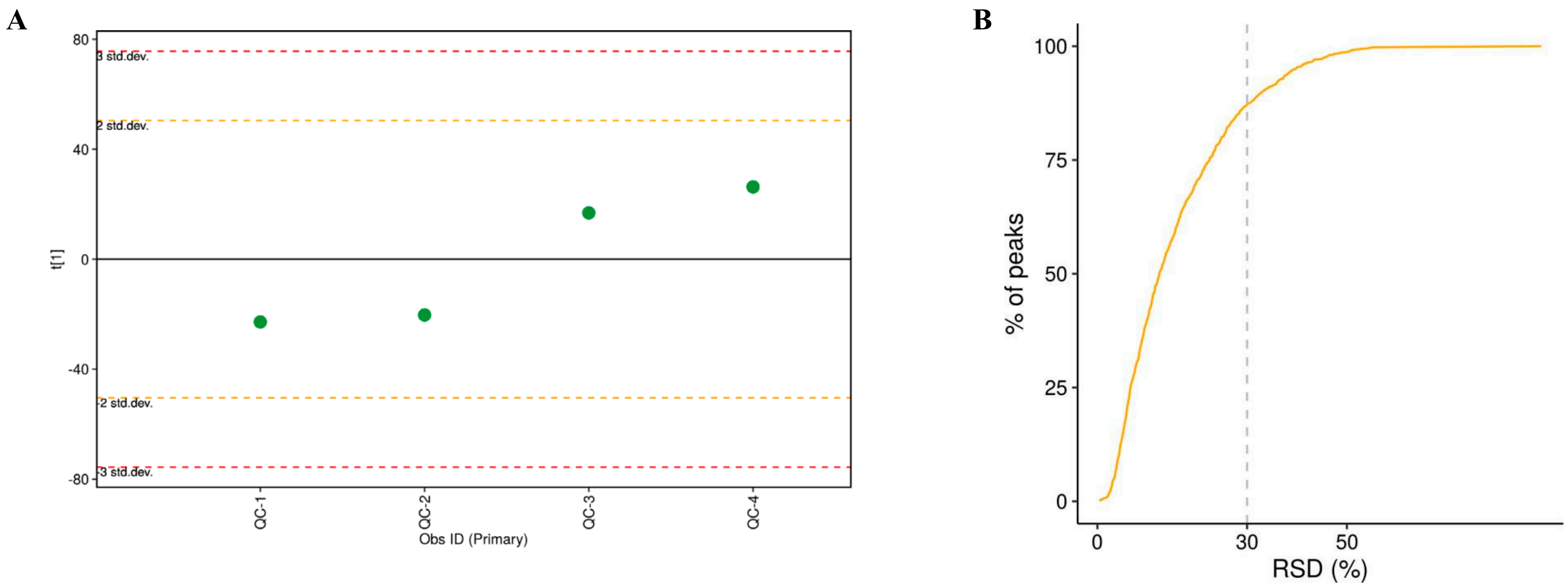

Figure S3: The reliability of data quality. The MCC diagram (A) and RSD (B) of QC samples.

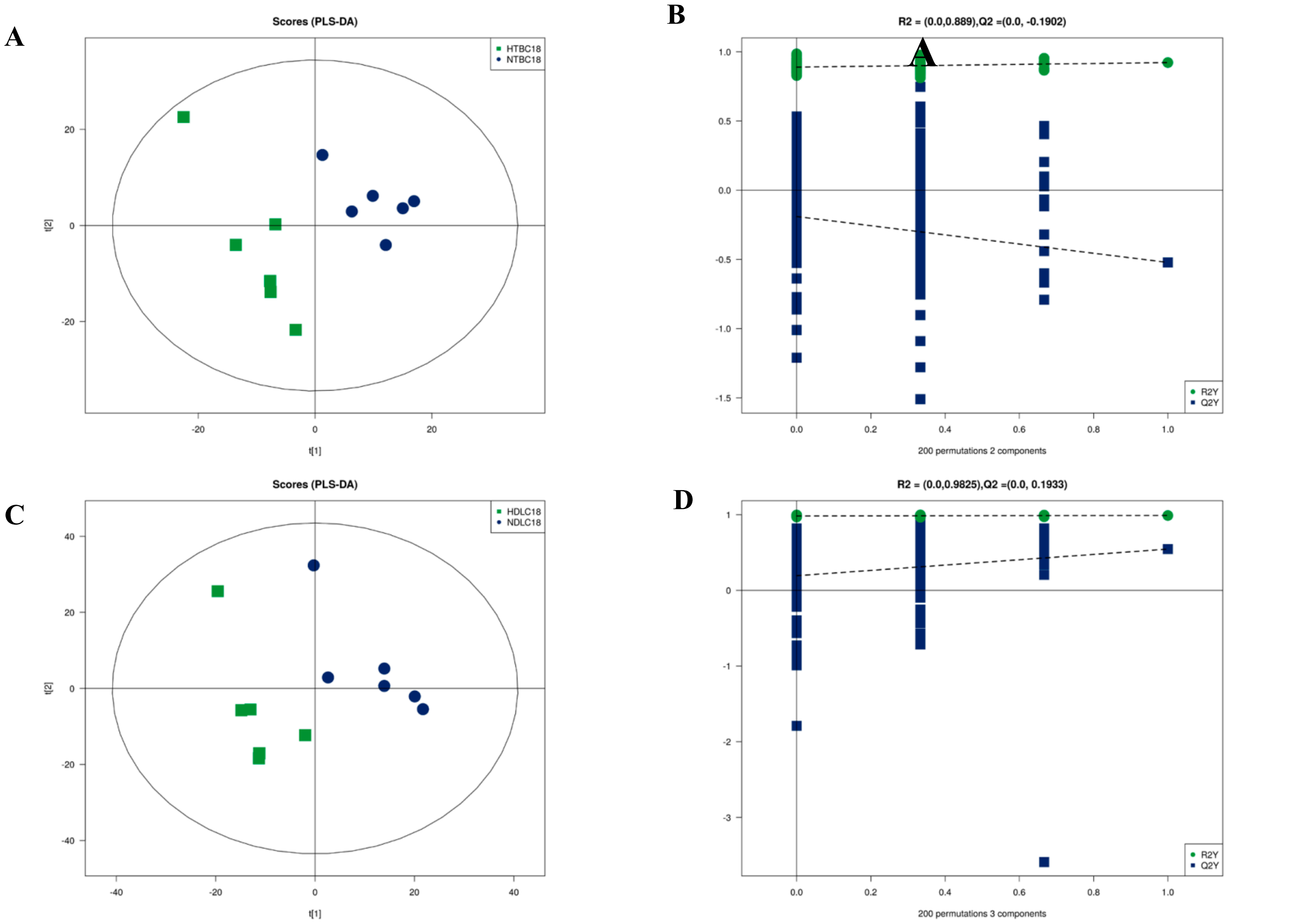

Figure S4: Multivariate analysis of lipidomics in TBCs and DLCs. PLS-DA score plot (A) and permutation test (B) between HTBC18 and NTBC18. PLS-DA score plot (C) and permutation test (D) between HDLC18 and NDLC18.

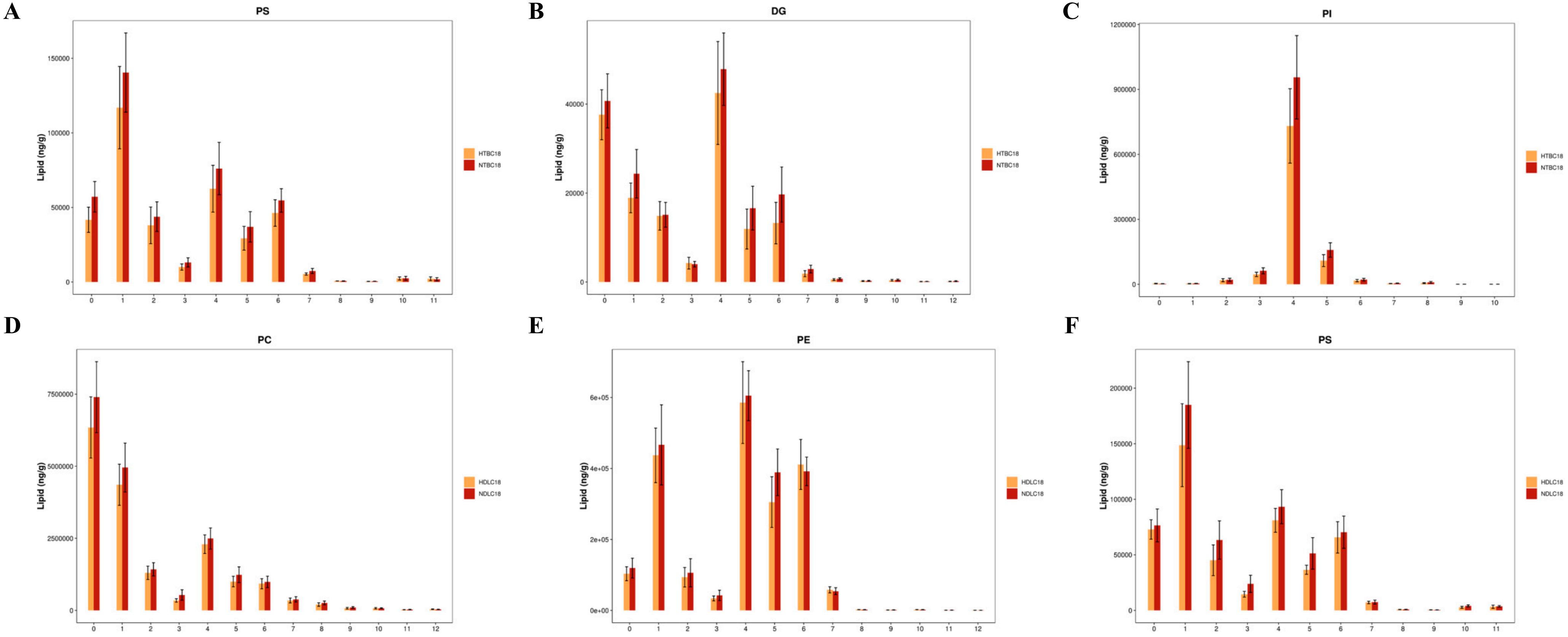

Figure S5: Lipid chain length analysis in HTBC18 vs NTBC18 (A-C) and HDLC18 vs NDLC18 (D-F).
